# The role of pparγ and autophagy in ros production, lipid droplets biogenesis and its involvement with colorectal cancer cells modulation

**DOI:** 10.1186/s12935-017-0451-5

**Published:** 2017-09-15

**Authors:** José Antonio Fagundes Assumpção, Kelly Grace Magalhães, José Raimundo Corrêa

**Affiliations:** Departamento de Biologia Celular, Instituto de Ciências Biológicas, Universidade de Brasília, Brasília, Brazil

**Keywords:** Colorectal cancer, Autophagy, PPAR, ROS, Lipid bodies, Cancer stem cells

## Abstract

**Background:**

In cancer cells, autophagy can act as both tumor suppressor, when autophagic event eliminates cellular contends which exceeds the cellular capacity of regenerate promoting cell death, and as a pro-survival agent removing defective organelles and proteins and helping well-established tumors to maintain an accelerated metabolic state while still dealing with harsh conditions, such as inflammation. Many pathways can coordinate the autophagic process and one of them involves the transcription factors called PPARs, which also regulate cellular differentiation, proliferation and survival. The PPARγ activation and autophagy initiation seems to be interrelated in a variety of cell types.

**Methods:**

Caco-2 cells were submitted to treatment with autophagy and PPARγ modulators and the relationship between both pathways was determined by western blotting and confocal microscopy. The effects of such modulations on Caco-2 cells, such as lipid bodies biogenesis, cell death, proliferation, cell cycle, ROS production and cancer stem cells profiling were analyzed by flow cytometry.

**Results:**

PPARγ and autophagy pathways seem to be overlap in Caco-2 cells, modulating each other in different ways and determining the lipid bodies biogenesis. In general, inhibition of autophagy by 3-MA leaded to reduced cell proliferation, cell cycle arrest and, ultimately, cell death by apoptosis. In agreement with these results, ROS production was increased in 3-MA treated cells. Autophagy also seems to play an important role in cancer stem cells profiling. Rapamycin and 3-MA induced epithelial and mesenchymal phenotypes, respectively.

**Conclusions:**

This study helps to elucidate in which way the induction or inhibition of these pathways regulate each other and affect cellular properties, such as ROS production, lipid bodies biogenesis and cell survive. We also consolidate autophagy as a key factor for colorectal cancer cells survival in vitro, pointing out a potential side effect of autophagic inhibition as a therapeutic application for this disease and demonstrate a novel regulation of PPARγ expression by inhibition of PI3K III.

**Electronic supplementary material:**

The online version of this article (doi:10.1186/s12935-017-0451-5) contains supplementary material, which is available to authorized users.

## Background

Colorectal cancer is the third most commonly diagnosed type of cancer in males and the second in females worldwide. Over 1.3 million of new cases, causing 694,000 deaths, have occurred in 2012 [1]. In 2015, was estimated 69,090 men and 63,610 women will be diagnosed with colorectal cancer and 26,100 men and 23,600 women probably will die of this disease only in the United States [2]. In particular, esophagus, stomach, and colon are hot spots in the digestive tract at high risk of developing cancer: indeed, esophageal, gastric, and colorectal cancers (CRC) represent very common malignancies disorders and account for approximately 30% of cancer-related deaths worldwide [3]. More than 90% of colorectal cancers are classified as adenocarcinoma, the lymphoma and squamous cell carcinoma are grouped in a cluster of rare malignancies of the gastrointestinal tract [4]. Therefore, research efforts on a better understanding of the pathogenesis initiation factors, therapeutic targets and potential biomarkers in CRC are still needed.

The etiology of CRC is still subject to scientific scrutinizing, as many different factors can contribute to its development. It is estimated that genetic syndromes and family history, together, may explain up to 30% of CRC susceptibility [5]. Although the genetic and epigenetic changes associated with the establishment of different gastrointestinal cancers were described in several recent studies [6, 7], lately, the key role of inflammation processes linked with the pathogenesis of colorectal cancer began to be described [8, 9]. The risk of developing CRC is significantly increased in people with inflammatory bowel diseases, such as ulcerative colitis and Crohn’s disease [10]. According to epidemiological studies, regular long-term use of anti-inflammatory drugs can reduce the mortality in groups of individuals with tumors at digestive tract [11]. Thus, the maintenance of the intestinal homeostasis also depends on the balance between tolerance and inflammation conditions, which involves a variety of cellular pathways. One of these pathways is autophagy, an intracellular process associated with the cell homeostasis regulation, innate immunity response and inflammation [12]. Pathogenesis such as Inflammatory Bowel Disease can be triggered by a slight deregulation on the autophagic process, which may result in tumor development [13]. Mutational events, which impair the autophagy pathways, have been shown to induce gastrointestinal problems, such as Crohn’s disease and increased risk of CRC development [14].

The interruption of the autophagic flux leads to an intracellular accumulation of organelles, protein aggregates and lipid droplets [15]. In many cases, the overall process of autophagy has both positive and negative roles in a given disease [16, 17]. Regarding cancer, autophagy has a dualistic role, functioning as a tumor suppressor and as a survival factor [18, 19]. It acts as a tumor suppressor removing dysfunctional organelles, which can lead to cellular stress and ultimately induce a chronic inflammation state [20]. As survival factor, autophagy allows cancer cells to generate new substrates for its maintenance and growth through recycling of “self” material, which aids tolerance to excessive stress [21–23].

Several different molecules can regulate the autophagic process. One of the most studied autophagic key regulators is the mammalian target of rapamycin (mTOR). This kinase protein is a convergence point for several pathways and is considered the most important negative regulator of autophagy [24, 25]. The mTOR protein integrates the upstream signals that regulate autophagy, cell proliferation and cell survival pathways through PI3K-Akt (phosphatidylinositol-3 kinase class I-B protein). The mTOR inhibition is an essential event for initiation of autophagosome formation [26]. Other important metabolism regulators recently related to the autophagic process are the peroxisome proliferator-activator receptors (PPAR), which were grouped as members of ligand-activated nuclear receptor proteins, including PPARα, PPAR β/δ and PPARγ. Upon ligand activation, PPARγ activates the transcription of target genes containing peroxisome proliferator responsive elements [27]. In cancer models, PPARγ activation decreases PI3K activity by modulating PTEN protein expression, suggesting its role in the regulation of the autophagic process as well [28–30].

The autophagic pathway regulation by PPARγ activation and the PPARγ expression modulation by autophagy in colorectal cancer cells have not yet been reported. Moreover, the combined effects of these pathways modulation simultaneously are unknown in this type of cancer cells, rendering pertinent the division of these pathways and analysis the consequences of their regulations on the inflammation establishment, maintenance and over the tumoral properties. In this work, we evaluated the involvement of autophagy and PPARγ with cell proliferation, cell death, lipid droplets formation, cell cycle, ROS production and cancer stem cells profile using colorectal cancer cells as a model.

## Methods

### Cell culture and treatment

Colorectal adenocarcinoma cell line Caco-2 cells were cultured as confluent monolayers in high-glucose (4.5 g/L) DMEM supplemented with 10% Fetal Bovine Serum (FBS), 100.000 units/mL penicillin, 100 mg/L streptomycin and maintained in a 5% CO_2_ humidified atmosphere at 37 °C. Cells were passaged when reaching 80–90% confluence.

### Autophagy and PPARγ modulation

Cells were treated with autophagy inducer, Rapamycin (Rapa) (100 nM), PI3K III inhibitor, 3-methyladenine (3-MA) (5 mM), PPARγ agonist, Rosiglitazone (Rosi) (10 µM), PPARγ antagonist, GW-9662 (GW) (10 µM) and combinations of these stimuli for 24 or 48 h. All stock solutions were prepared in DMSO and the samples incubated in media solution containing the corresponding diluents volumes were used as negative controls.

### Western blotting analyses

After treatments, the samples were rinsed three times in phosphate buffered saline (PBS) and lysed with cell lysis buffer (50 mM Tris–HCl, 150 mM NaCl, 5 mM EDTA, 1% Triton X-100) at pH 7,4 containing proteases inhibitor. The cell lysates were kept on ice and vigorously vortexed each 5 min, for 30 min, and the pellet was collected after centrifugation (15,000 rpm, 10 min, 4 °C). The protein concentrations were determined using the Bradford Assay. The proteins were separated by SDS-PAGE on a 10% gradient acrylamide gel and transferred to nitrocellulose membrane. The membranes were blocked for 1 h at room temperature (RT) with 5% (w/v) non-fat dry milk in Tris buffer saline (TBS) with 0.05% (v/v) of Tween20 (TBST). The membrane was incubated overnight at 4 °C with the primary antibodies, rabbit anti-MAP LC3β H-50 (SC-28266) and rabbit anti-PPARγ H-100 (SC-7196). The membrane was washed and incubated for 1 h with the appropriate horseradish peroxidase-conjugated anti-rabbit antibody. The signals were revealed by electrochemoluminescence using the Image Quant LAS 4000 Series and then quantified by pixel densitometry using the ImageJ software.

### Immunofluorescence assay and lipids droplets localization

Following treatment for 24 h, samples were rinsed three times in PBS and fixed overnight in formalin 3.7% at RT. The cells were permeabilized using 0.2% Triton X-100 for 20 min. Samples were blocked in 2.5% BSA for 20 min at RT. For PPARγ detection, it was used the anti-PPARγ H-100 and to neutral lipids localization the cells were stained with BODIPY. The secondary antibody conjugated with Alexa Fluor 546 or Alexa Fluor 633 was used to detect the primary antibodies. The nuclei were stained using DAPI as recommended by its manufacturer. The images were acquired using a laser scanning confocal microscope Leica TCS SP5.

### Acridine Orange staining of acidic vesicular organelles (AVOs)

Autophagy is characterized by AVOs formation. To detect intracellular acidic vesicles, Acridine Orange (AO) was used. AO is a weak base that accumulates on autophagosomes and lysosomes due to the low lysosomal pH. After treatment, the cells were incubated with AO (1 µg/mL) for 15 min at 37 °C protected from light. The cells were washed and resuspended in PBS for flow cytometry analysis.

### Lipid bodies’ biogenesis assay

Treated cells were fixed with 3.7% formalin for 10 min and washed with Milli-Q water. The samples were incubated with 100% propylene glycol for 2 min and posteriorly with 0.5% Oil Red O solution for 15 min. The samples were washed three times in 60% propylene glycol for 1 min and with Milli-Q water. The nuclear staining was achieved using Hematoxylin for 5 s, followed by Milli-Q water rinsing. The microscope slides were mounted using Entellan.

### Cell death assay

After treatments, the samples were washed with PBS, resuspended in 100 µL of Annexin-V Binding Buffer 1× and incubated with 2.5 µL of Annexin-V FITC for 15 min, at RT, protected from light. The samples were incubated in Propidium Iodide solution (2 µg/mL in Annexin-V Binding Buffer for 5 min at 4 °C protected from light. The samples were immediately analyzed by flow cytometry. As experimental control, apoptosis was induced using 20% DMSO for 15 min and necrotic cells were obtained heating the cells at 70 °C for 15 min.

### Cell proliferation assays

Following treatments, the cells were rinsed three times in PBS and incubated with 10 µM CFSE for 15 min at 37 °C protected from light. The samples were washed three times in PBS and complete culture medium was added for 30 min. It was used cells treated with 10 µM Colchicine as experimental control. The samples were also staining by crystal violet as an alternative analysis method of cell proliferation. Cells treated for 24 and 48 h were fixed with ethanol for 10 min and stained with 0.05% crystal violet in 20% ethanol for 10 min. After the dye solubilization with methanol, the plate was read in a microplate reader (595 nm).

### Cell cycle analyses

Treated cells were resuspended in 100 µL of PBS and it was added 900 µL of ice-cold ethanol 70% and kept for 2 h at 4 °C. The samples were centrifuged at 5000 rpm for 10 min and the pellet was washed three times in PBS and resuspended in a standard staining solution (0.1% Triton X-100, 100 µg/mL PI and 50 µg/mL DNAse-free RNAse) for 10 min at 37 °C protected from light. The samples were resuspended in PBS for flow cytometry analysis.

### Fluorescence analysis of reactive oxygen species (ROS)

After treatment, the cells were incubated with 5 µM CellROX for 30 min at 37 °C protected from light. After staining, the samples were washed three times in PBS and resuspended in same buffer for analysis in flow cytometer. As an experimental control, the cells were treated with 10 µM Rotenone.

### Analysis of CD44+/CD24− expression

Following treatment, the samples were rinsed three times in 1% BSA/PBS and incubated with anti-CD44-FITC and anti-CD24-PE antibodies in blocking solution (1% BSA/PBS), at final concentration of 1:20 for 30 min at 4 °C. The samples used as negative control were incubated with corresponding antibody isotypes.

### Statistical analysis and gating strategy

The data were expressed as mean ± SD from at least three separate experiments performed in triplicates. Differences between groups were analyzed using Student’s t test and the p < 0.05 were considered statistically significant.

For all flow cytometry assays, the gating was performed using morphologic parameters, forward scatter (FSC) and side scatter (SSC) to select the most uniform population containing viable cells.

## Results

### PPARγ modulators can regulates autophagy in Caco-2 cell line

**Fig. 1:**
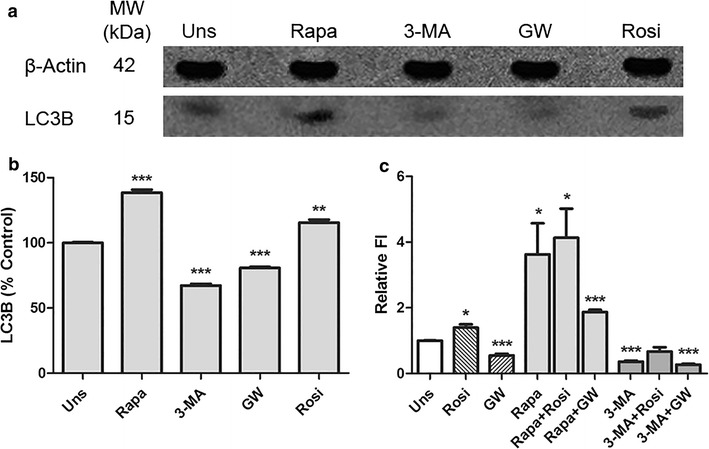
PPARγ regulates autophagy in Caco-2 cell line. **a**, **b** Western blot result to LC3B expression in Caco-2 cells after 24 h’ treatments. It was used beta-actin as internal control. Data represents three independent experiments. **p < 0.005, ***p < 0.0001. **c** The AVOs quantification by Acridine Orange staining. The relative fluorescence intensity was normalized by the unstimulated control. *p < 0.05, ***p < 0.001

It was first analyzed the autophagic status of Caco-2 colorectal cancer cell line treated with PPARγ agonist and antagonist. To address this issue, the expression of LC3B (a marker and specific constituent of the autophagosome membrane) was investigated. It was demonstrated by Western blot analysis an increase of LC3B expression after administration of Rapamycin and Rosiglitazone. In contrast, the use of 3-MA and GW-9662 caused a decrease in the LC3B expression when compared to the unstimulated control (Fig. 1a, b).

To corroborate the results obtained through immunoblotting, the formation of AVOs was quantified. The results of the sampling of autophagic process were highly concordant. Rapamycin and Rosiglitazone increased the number of AVOs, whereas 3-MA and GW-9662 reduced them. The co-stimulation yielded no significant difference between treatments (Fig. 1c).

### Autophagy can regulate PPARγ expression in Caco-2 cell line

**Fig. 2:**
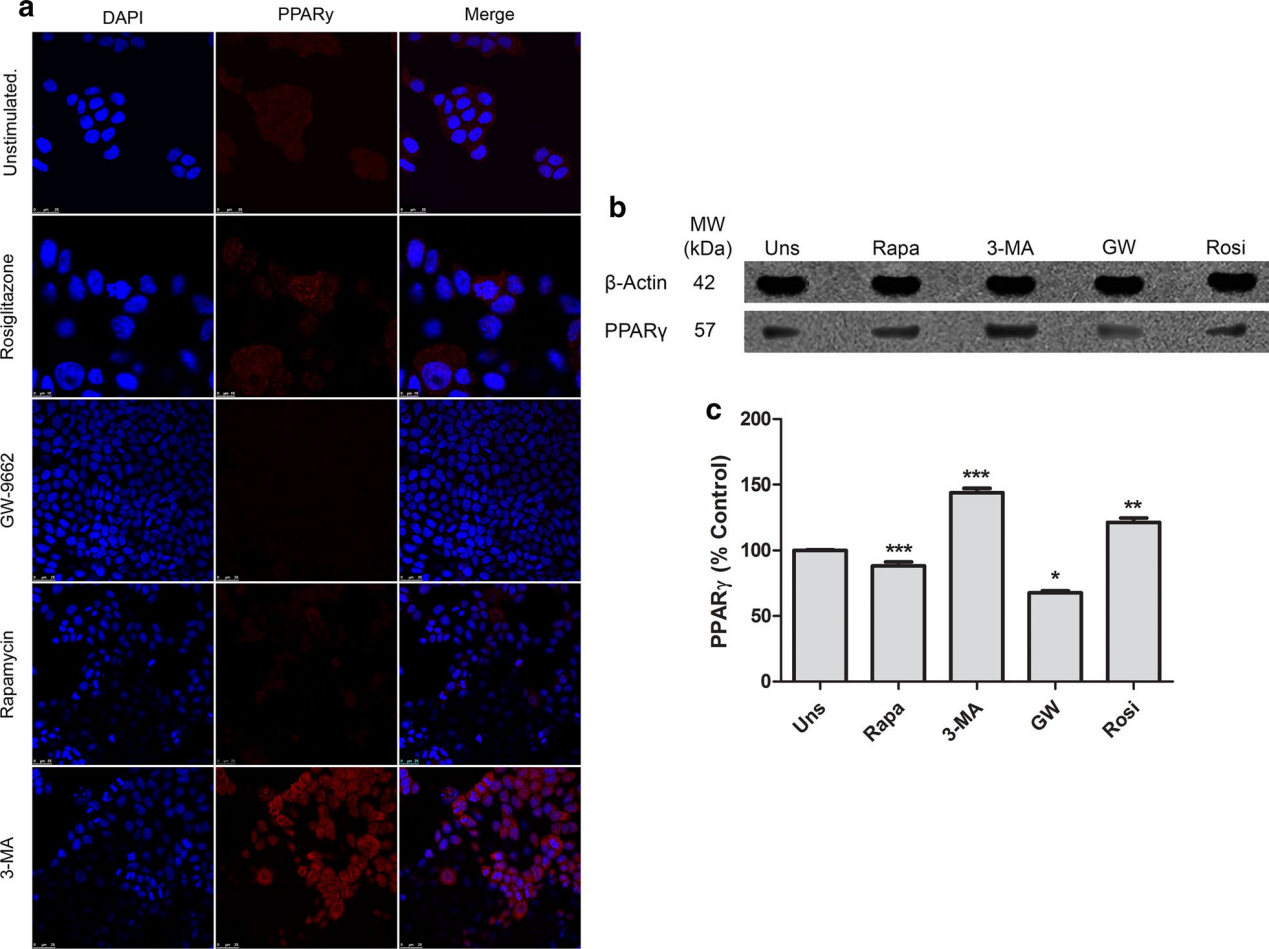
Autophagy regulates PPARγ expression in Caco-2 cell line. **a** Representative of confocal microscopy of Caco-2 cells, PPARγ (red) and DNA (blue). Reference scale bar: 25 µm. **b**, **c** The Western blot result to PPARγ expression in Caco-2 cells after 24 h’ treatments. It was used beta-actin as internal control. This data represents three independent experiments. *p < 0.05, **p < 0.005, ***p < 0.001

To evaluate a possible regulation of PPARγ by the autophagic process, we analyzed PPARγ expression in Caco-2 cells after treatment with Rapamycin and 3-MA. Our results suggest a constitutive PPARγ expression in Caco-2 cell line and that the treatment with Rapamycin caused no effect on its expression. The autophagic inhibition by 3-MA, however, induced high expression of PPARγ. The cells treated with Rosiglitazone and GW-9662 showed a slight modification of PPARγ expression profile. The PPARγ expression was induced or repressed respectively, as expected (Fig. 2a). The quantitative analysis of PPARγ expression pointed to increased expression in the presence of PPARγ agonist, rosiglitazone, and a higher expression of PPARγ after 3-MA treatment. The addition of PPARγ antagonist GW-9662 and Rapamycin resulted in a decrease in its expression (Fig. 2b, c).

### The inhibition of autophagy and PPARγ increased the number of lipid bodies in Caco-2 cell line

**Fig. 3:**
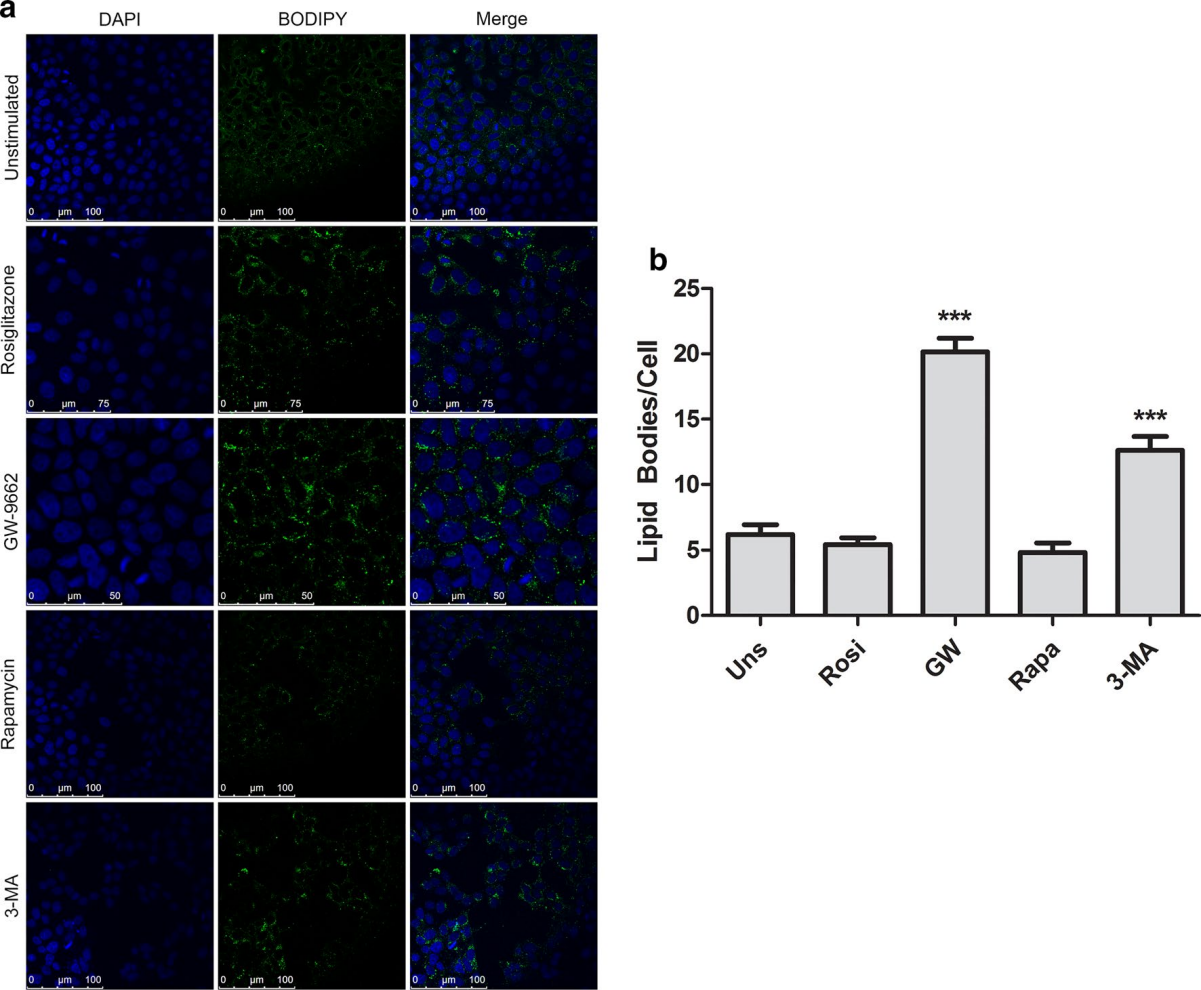
Inhibition of autophagy and PPARγ increases the number of lipid bodies in Caco-2 cells. **a** A representative confocal microscopy of Caco-2 cells. The lipid bodies were stained in green and DNA in blue. **b** The lipid bodies quantification per cell after treatments. This data represents three independent experiments. ***p < 0.0001

To verify the association among autophagy, PPARγ expression and lipid bodies accumulation, it was analyzed the lipid bodies’ biogenesis in Caco-2 cells. The cells treated with Rosiglitazone, Rapamycin or unstimulated cells, did not show any statistic difference in number of lipid bodies (Fig. 3a). The treatment with 3-MA and GW-9662 increased the cells biogenesis and accumulation of lipid bodies. Interestingly GW-9662 induced the cells to produce a greatest number of these organelles, in an average of 12–20 lipid bodies per cell (Fig. 3b).

### The activation of PPARγ and the inhibition of autophagy in Caco-2 cell line, reduced the cell viability and induced cell death by apoptosis

Since a positive relationship between the autophagic process and the expression/activation of PPAR could be found, we begun to analyze the effect of its modulation on cellular properties, which is close associated with the tumor maintenance, starting by cell viability and cell death profiles.

**Fig. 4:**
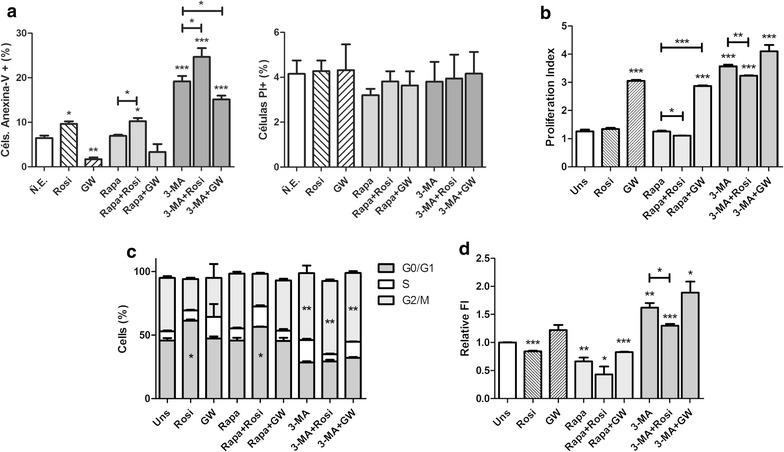
Effects of autophagy and PPARγ modulation in Caco-2 cells. **a** Activation of PPARγ and inhibition of autophagy reduce the cell viability and induce cell death by apoptosis in Caco-2 cells. **b** Changes in Caco-2 cells proliferation index. **c** Caco-2 cells’ cycle profiling after modulation (percentages of cells in G0/G1, S and G2/M phases are indicating in the graph according to the graphic legend). **d** Changes in ROS production in Caco-2 cells (relative fluorescence intensity was normalized to unstimulated control). All data represents three independent experiments. *p < 0.05, **p < 0.01, ***p < 0.001

Interesting, the decrease in cell viability observed in samples treated with rosiglitazone or GW-9662 could be explain by different profile of cell death induction. The rosiglitazone induced cell death mainly by apoptosis while the GW-9662 induced cell death mostly by necrosis (Fig. 4a). Regarding to the autophagic modulation, only the autophagy inhibition caused by 3-MA treatment resulted in a significant reduction of cell viability with correlate increases of cells in apoptosis (Fig. 4a). The combined use of Rosiglitazone and 3-MA resulted in no significant difference of cell death profile. The samples treated with GW-9662 and 3-MA combined showed an increased number of viable cells with correlate reduced number of apoptotic cells, compared with the samples treated only with 3-MA (Fig. 4a). All treatment using 3-MA promoted a significant reduction of cell viability. None of the treatments substantially affected the cell membrane integrity as show by PI permeability results (see Additional file 1).

### Changes in proliferative capability of colorectal cancer cells can be induced by autophagic modulation and PPARγ activation

It was observed significant differences in cell proliferation only in the samples treated with GW-9662 and 3-MA, both compounds caused an increase in cell proliferation index, i.e., the average number of cell divisions. The use of Rosiglitazone produced a slight decrease in cells proliferation when used in conjunction with Rapamycin and when compared with proliferation index obtained from single treatment with Rapamycin (Fig. 4b).

To corroborate the results obtained by CFSE, it was performed additional cell proliferation analysis using Crystal Violet for 0, 24 and 48 h. The results from Crystal Violet assay strong corroborated the previously data obtained in CFSE assay (see Additional file 1).

### Cell cycle analysis of Caco-2 cells after modulation of autophagy and PPARγ

Changes in cell survival and proliferation are often related to changes in the cell cycle. The samples treated with Rosiglitazone and 3-MA alone or combined produced statistical significant modifications on cell cycle phases. Rosiglitazone caused cell cycle arrest in G0/G1 phase, while 3-MA caused interference with cells in S and G2/M phases (Fig. 4c). These results support and help to explain the data obtained previously in the apoptosis assay.

### Association among autophagy, PPARγ modulators and reactive oxygen species (ROS) production

All signaling pathways linked to ROS production are very important to cancer cells. In cancer cells, the ROS signals can lead these cells to a series of different outcomes, which include the cell death. The stimulation with Rosiglitazone caused a decrease in the levels of intracellular ROS, whereas stimulation with GW-9662 showed no significant difference. Rapamycin and 3-MA resulted in decreased and increased levels of ROS, respectively. Except for the combined use of 3-MA and Rosiglitazone, which reduced the amount of ROS compared to stimulation of 3-MA alone, the other co-stimulation showed no significant differences (Fig. 4d). The increased ROS production in 3-MA treated cells is also in agreement with cell death results obtained previously.

### Cancer stem cells profiling in Caco-2 cells after PPARγ and autophagy modulation

**Fig. 5:**
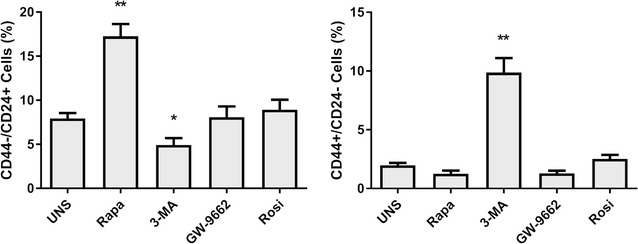
Autophagic modulators alter the profile of Caco-2 cancer stem cells. Data represents three independent experiments. *p < 0.05, **p < 0.01, ***p < 0.001

One of the fundamental capabilities of cancer cells is their plasticity, which allows these cells to adapt to novel conditions in response to environmental changes. Sometimes, these aspects can influence the phenotype of specific cells subpopulation, making them acquire more epithelial or mesenchymal characteristics. The stimuli related to PPARγ activity gave by Rosiglitazone and GW-9662, did not change the profile of tumor stem cells, translated by the absence of significant change in the percentage of this cell population, measured by the balance between CD24−/CD44+ expression. Differently, the samples treatment with autophagic modulators altered the profile of tumor stem cells (Fig. 5). Rapamycin was able to induce its displacement to an epithelial phenotype (CD44−/CD24+) and 3-MA lead these cells to a mesenchymal phenotype (CD44+/CD24−).

## Discussion

There are many conjectures whether the pathways linked to autophagy and those activated by PPARγ are connected and related to cancer cell survival and colorectal cancer tumorigenesis [31–34]. Their key role in cancer development make the details of their modulation an interesting target for potential drug development.

Our results showed that PPARγ activation can induce autophagy and its inhibition decreases it, consistent with findings by other groups [35]. Co-stimulation with Rapamycin and 3-MA did not significantly alter the number of AVOs, implying autophagy does not necessarily require PPARγ activation. PPARγ activation by Rosiglitazone causes positive regulation of the phosphatase and tensin homolog gene (PTEN) in Caco-2 cells and other cells types [36]. PTEN regulates a variety of cellular functions, including migration, survival and proliferation [37], mainly antagonizing the signaling cascade mediated by PI3K. Thus, PI3K-Akt-mTOR pathway inhibition could initiate the autophagic process. Failure in observing differences in co-stimulation treatments may be due to the fact that the stimuli administered act downstream to PPARγ and PTEN [38, 39].

In the opposite direction, regulation of PPARγ expression by autophagy modulation was not observed in cells treated with Rapamycin, suggesting mTOR and the induction of autophagy do not cooperate to PPARγ modulation. PI3K III blocking by 3-MA treatment caused a dramatic increase in PPARγ levels. Translocation of PPARγ from cytoplasm to the cells nuclei, however, was not observed. There is no record of PPARγ interaction or regulation by PI3K III, remaining unknown the intracellular signaling responsible for the results demonstrated. High protein levels could lead to protein aggregates initiating autophagy, since the need to be removed from cells’ cytoplasm, but there is lack of evidence to support this hypothesis [40].

Interestingly, in the present work, lipid bodies’ biogenesis observed in Caco-2 cells differs from previously described [41, 42]. According to our data, PPARγ inhibition increases the lipid bodies’ biogenesis in cells treated with GW-9662. Treatment with 3-MA resulted in a significant increase in the number of lipid bodies per cell. The regulation of fatty acid uptake and lipid storage are usually related with PPARγ translocation into the nucleus [43], not observed in our work. There are at least two plausible explanations: First, a process responsible for the degradation of lipid stores, called lipophagy, which could regulate the number of lipid bodies in Caco-2 cells [44]. Since both stimuli (GW-9662 and 3-MA) resulted in decreased levels of autophagy, the high number of lipid bodies may be associated with reduced lipophagy. Secondly, a positive relationship between autophagy inhibition and ROS production was observed, and there are evidences linking ROS production and lipid droplets formation [45]. Autophagy inhibition can also reduce the mitochondria defective removal from these cells, leading to accumulation of ROS and triggering lipid droplets biogenesis [46, 47]. Furthermore, to avoid excessive oxidative stress, cells can limit the amount of lipids -oxidation by storing fatty acids in organelles (i.e. lipid droplets) as a survival mechanism [48].

**Fig. 6:**
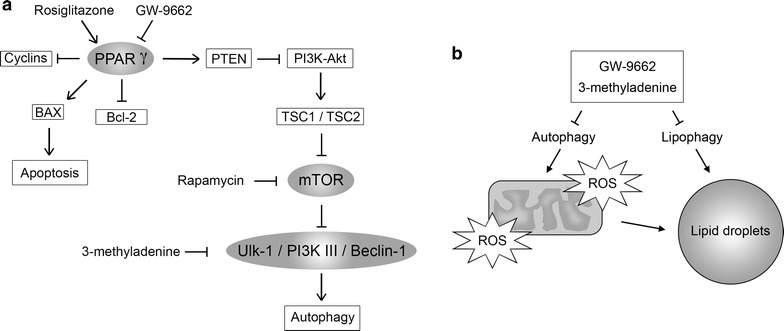
Model to autophagy and PPARγ pathways overlapping and regulating each other, which induce the lipid droplets biogenesis. **a** Suggests interaction and control of the pathways linked to PPARγ and autophagy. **b** A probably mechanism of lipid droplets biogenesis induction

Combining our data, we proposed a model, Fig. 6, suggesting the interaction and control of the pathways linked to PPARγ and autophagy as well a possible mechanism to lipid droplets biogenesis.

The last part of this study was addressed to help us understand how the activation or inhibition of the autophagic pathway and PPARγ might interfere with specific tumor properties, such as survival, proliferation and aggressiveness [49, 50]. Other studies have raised the possibility of using these pathways as markers for colorectal cancer [51, 52]. There is, however, no reporting about the combined action of these pathways in cancer progression.

In Caco-2 cell line, cell death by apoptosis was induced by PPARγ activation and autophagic inhibition. Treatment with 3-MA lead up to 40% of cells to apoptosis, reaffirming autophagy as a fundamental pathway in colorectal cancer survival, promoting stress tolerance, organelles renewal and increased energetic availability [53, 54]. In colon cancer cells, treatments with PPARγ ligands usually induce antineoplastic effect. The co-stimulatory results, however, indicate that PPARγ and autophagy influence cell death by different signaling pathways. Their combined impact on cell death is still not clear.

Regarding sustained proliferative signaling, our results suggest PPARγ inhibition increases cell proliferation, in agreement with other studies [55]. Autophagic inhibition, contradicting the results obtained from cell death assays, induced cell proliferation in Caco-2 cells. PI3K III is found downstream of mTOR, core protein in a variety of metabolic pathways [56]. One possible explanation for the increased cell proliferation is the activation of an unknown compensatory mechanism due to blockage of PI3K III by 3-MA. Upregulation of mTOR activity results in advantages in cell growth, proliferation and survival [57]. Compensatory mechanisms involving mTOR-regulated pathways already have been described, including studies about antitumor therapies [58, 59].

Another hypothesis proposed by us, is that this modulation is due to an effect called apoptosis-induced proliferation. Apoptotic cells can stimulate increased cell proliferation in surviving neighboring cells [60, 61]. Since the analysis of cell proliferation is done only in living cells, it is possible to state that the increased cell proliferation observed in cells treated with 3-MA can be a response to a stimulus received from nearby apoptotic cells.

Proliferative variations were not necessarily caused by alterations in cell cycle progression. Highlighting the antitumor and apoptosis-inducing effects of Rosiglitazone, a slight retention of the cell cycle in G0/G1 phases was observed, consistent with the suppression of Cyclin D1 expression described in previous studies [62]. 3-MA stimulation resulted in cells arrest in G2/M phase of cell cycle, suggesting the non-removal of cellular elements by autophagy may affect chromosome segregation. Unexpectedly, the combination of 3-MA and Rosiglitazone reversed the effect observed for Rosiglitazone alone, but the cells’ retention in G2/M phases remains.

The inflammatory state preceding tumor development is thought to contribute to the generation of dysplastic lesions through ROS production, inducing excessive cell damage [63]. Treatments for colorectal cancer use ROS inducers, usually demonstrating pro-apoptotic effects [64]. In similar ways, 3-MA caused both increases in ROS production and in cell death induction. Curiously, Rosiglitazone reduced intracellular ROS, indicating that PPARγ activation-induced apoptosis is related to other signaling pathways (previously discussed). Autophagy also exerts control over ROS levels, through a negative feedback mechanism [65]. Inhibition of autophagy can generate, therefore, higher levels of ROS and lead to apoptosis.

We also investigated whether autophagic and PPARγ-mediated pathways could modify a phenotype aspect linked to cancer stem cells (CSCs) population, measured by CD24/CD44 differential expression. This subset of cells undergoes self-renewal, differentiation and gives rise to all cancer cells type in a tumor, as a normal stem cell would do. It also dictates the metastatic capability and therapy resistance in tumors, making the identification of CSCs surface markers and the development of CSC-targeted treatments an essential subject for cancer research [66, 67]. Here, we demonstrated that PPARγ modulation in Caco-2 cells does not alter CD44/CD24 expression patterns. However, the regulation of autophagic route showed significant differences in expression of these markers. Treatment with Rapamycin induced a CD44−/CD24+ phenotype, while the use of 3-MA led to CD44+/CD24− phenotypes.

CD44+/CD24− phenotype indicates cells have mesenchymal characteristics, like greater phenotypic plasticity, higher proliferation rate and lower expression of adhesion proteins, contributing to a higher metastatic capability, directly related with poor outcome for patients [68]. Thus, we can assume that this disease, in its terminal phase, has a larger population of tumor stem cells, which may have settled, at least partially, through the inhibition of autophagy by mechanisms not yet described. To the other hand, CD44−/CD24+ cells (induced by Rapamycin) indicate CSC with epithelial characteristics, with higher expression of adhesion proteins, and lower phenotypic plasticity, related to a global less aggressive cancer phenotype [69, 70]. Our results suggest that CRC treatment based on drugs that potentially inhibit autophagy, such as 3-MA, bafilomycin A1, and chloroquine, used to sensitize CRC to chemotherapy [71], can be harmful to the patients, inducing CSCs to phenotype CD44+/CD24−.

## Conclusions

Finally, in this work, the results obtained with autophagy pathway modulation, especially with the use of class III PI3K inhibitor, 3-MA, which generated an increase in the PPARγ expression, indicating a probable new mechanism controlling the expression of this nuclear transcription factor. Moreover, once again the control of autophagy in colorectal cancer was consolidating as a key factor in tumor survival. Our in vitro studies showed that autophagy inhibition or induction have potential clinical implications and these data could be applied to improve CRC therapeutic approaches. Most current studies support the idea of autophagic inhibition as potentiating of chemotherapy action against colorectal cancer by preventing the cytoprotective autophagy action. This work, however, emphasizes that, although cell death was higher in Caco2 cells with compromised autophagic flux, surviving CSC could acquire mesenchymal features as a “side effects” due to autophagy inhibition. These types of the cells are related with higher tumor aggressiveness and poor outcome to the patients. Further studies are essential to a deeper understanding of the relationship between these pathways, tumorigenesis and tumor maintenance. It is also extremely relevant to address the association of this modulation and its effect on cancer cells populations.

## Additional file

**Additional file 1.** Proliferation assay using Crystal Violet Staining, representative flow cytometry plots of cells stained with Annexin-V FITC and Propidium Iodide (PI) and representative flow cytometry plots of CD44/CD24 staining.

## Abbreviations

3-MA: 3-methyladenine
Akt: protein kinase B
AO: acridine orange
AVO: acidic vesicular organelle
CRC: colorectal cancer
CSC: cancer stem cell
FSC: forward scattering
GW: GW-9662, 2-chloro-5-nitrobenzanilide
LC3: microtubule-associated protein 1 light chain 3
mTOR: mammalian target of rapamycin
PPAR: peroxisome proliferator-activated receptor
PTEN: phosphatase and tensin homolog gene
Rapa: rapamycin (sirolimus)
ROS: reactive oxygen species
Rosi: rosiglitazone
RT: room temperature
SSC: side scattering
