# Correction to: The role of pparγ and autophagy in ros production, lipid droplets biogenesis and its involvement with colorectal cancer cells modulation

**DOI:** 10.1186/s12935-017-0463-1

**Published:** 2017-11-02

**Authors:** José Antonio Fagundes Assumpção, Kelly Grace Magalhães, José Raimundo Corrêa

**Affiliations:** Departamento de Biologia Celular, Instituto de Ciências Biológicas, Universidade de Brasília, Brasília, Brazil

## Correction to: Cancer Cell Int (2017) 17:82 DOI 10.1186/s12935-017-0451-5

Upon publication of the original article [1], it was noticed that corresponding author status has been erroneously attributed to José Antonio Fagundes Assumpção. The corresponding author of this manuscript should be noted as José Raimundo Corrêa (correa@unb.br).

This error has since been acknowledged and corrected in this Correction.
