# Supplementary material for: The role of pparγ and autophagy in ros production, lipid droplets biogenesis and its involvement with colorectal cancer cells modulation
Source: Cancer Cell Int. 2017 Sep 15;17:82. doi: 10.1186/s12935-017-0451-5 (PMC5603033; doi:10.1186/s12935-017-0451-5)
Supplement: Supplementary file 1 — Additional file 1. Proliferation assay using Crystal Violet Staining, representative flow cytometry plots of cells stained with Annexin-V FITC and Propidium Iodide (PI) and representative flow cytometry plots of CD44/CD24 staining. [file 12935_2017_451_MOESM1_ESM.pdf]

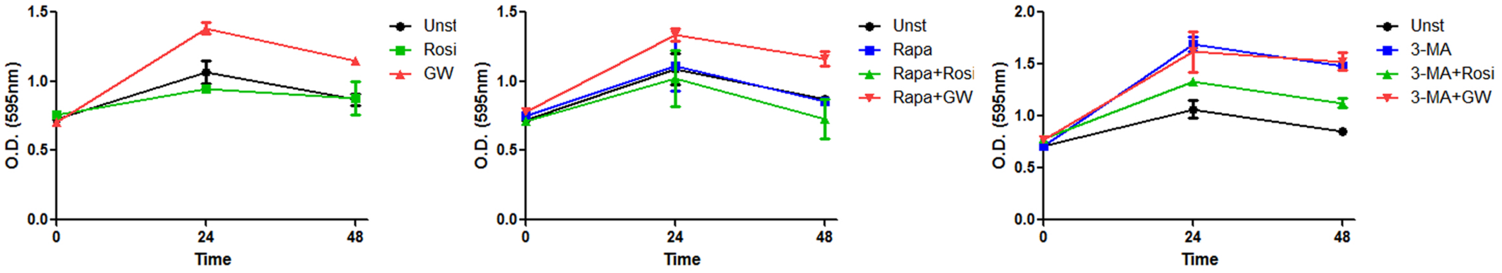


Supplemental Figure 1. Proliferation assay using Crystal Violet staining. Cells were treated for up to 48 hours prior analysis. The optical density was measured in a spectrophotometer at 595nm.

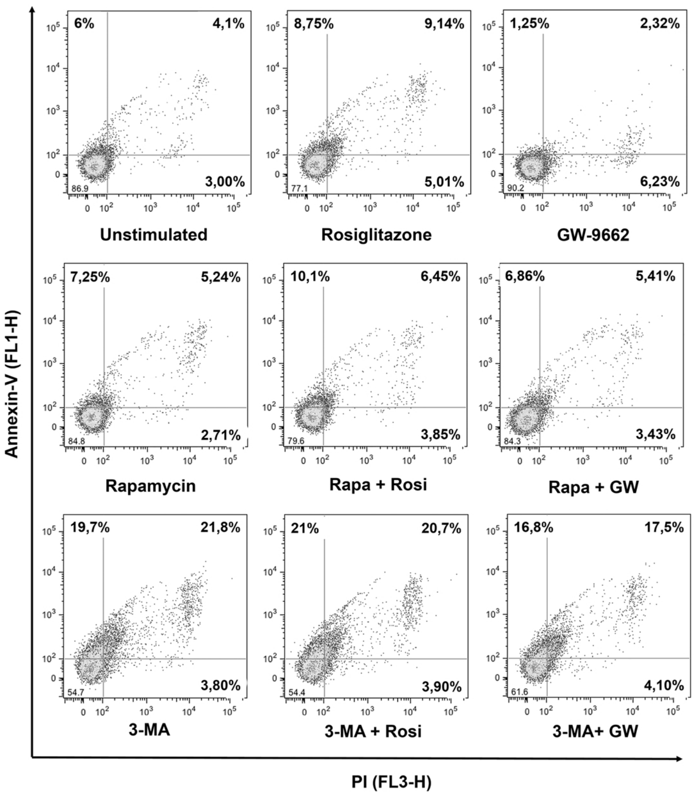


Supplemental Figure 2. Representative flow cytometry plots of cells stained with Annexin-V FITC and Propidium Iodide (PI). Numbers in quadrants represent percent of Annexin-V negative/-PI-negative cells (bottom left), Annexin-V positive/PI-negative cells (top left), Annexin-V positive/PI-positive cells (top right) and Annexin-V negative/PI-positive cells (bottom right). This data represents three independent experiments.

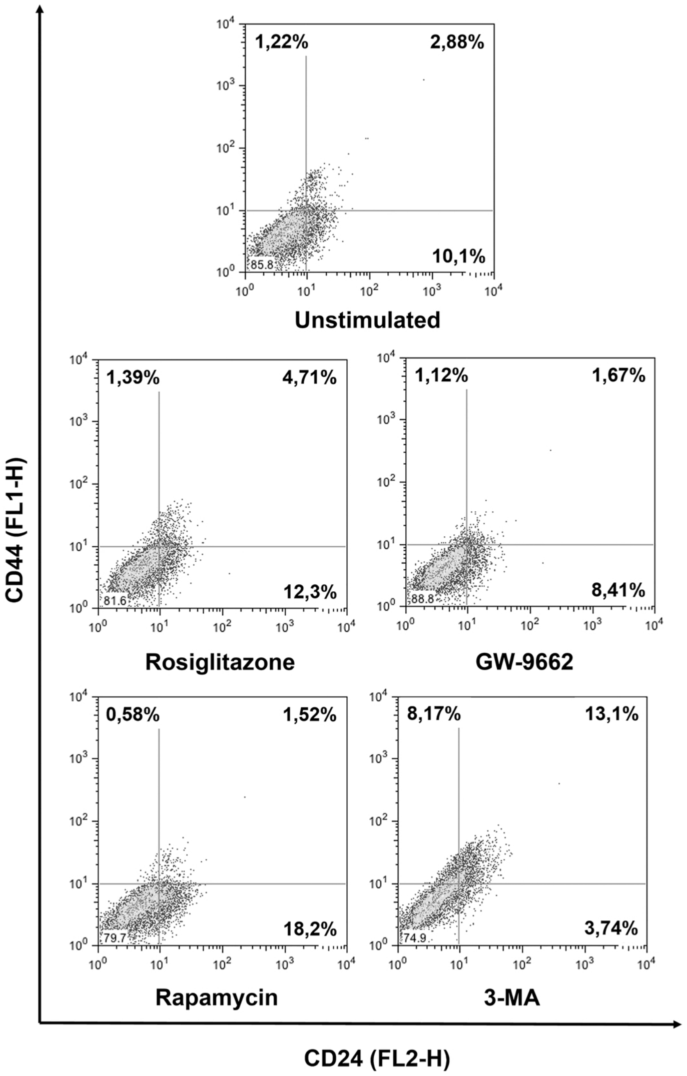


Supplemental Figure 3. Autophagic modulators alter the profile of Caco-2 cancer stem cells. Numbers in quadrants represent percent of CD44-negative/CD24-negative cells (bottom left), CD44-positive/CD24-negative cells (top left), CD44-positive/CD24-positive cells (top right) and CD44-negative/CD24-positive cells (bottom right). Data represents three independent experiments.
